# Application of artificial intelligence in rabbit husbandry: from reproductive monitoring to precision farming

**DOI:** 10.3389/fvets.2025.1679630

**Published:** 2025-10-24

**Authors:** Ahmed A. A. Abdel-Wareth, Ahmed A. Ahmed, Md Salahuddin, Jayant Lohakare

**Affiliations:** ^1^Cooperative Agricultural Research Center, College of Agriculture, Food and Natural Resources, Prairie View A&M University, Prairie View, TX, United States; ^2^Department of Animal and Poultry Production, Faculty of Agriculture, South Valley University, Qena, Egypt; ^3^Computer Science Department, Prairie View A&M University, Prairie View, TX, United States

**Keywords:** artificial intelligence, rabbit farming, parturition prediction, health monitoring, machine learning

## Abstract

Artificial Intelligence (AI) is revolutionizing animal husbandry by automating and optimizing management processes, with significant potential in rabbit farming. Due to rabbits’ high reproductive rates and diverse uses, AI can greatly improve reproductive management, health monitoring, and behavior analysis, as well as reproductive monitoring, health assessment, and precision farming. AI technologies, such as machine learning (ML), computer vision, and sensor integration, enable more efficient pregnancy detection, parturition prediction, delivery monitoring, and health surveillance. These systems innovations reduce reliance on manual labor, minimize monitoring time, and enhance animal welfare. AI-powered systems can detect pregnancy and predict parturition by analyzing physiological data, while wearable sensors and machine learning models monitor real-time health data information to identify early signs of illness. Additionally, AI tools track rabbits’ behavior, activity levels, and social interactions, ensuring optimal living conditions and reducing stress or injury. However, challenges remain, including data collection, ethical concerns, and adapting systems to diverse farming environments. Despite these obstacles, AI offers substantial benefits, including precision management, increased productivity, and improved animal wellbeing. As AI technologies evolve, further advancements in accessibility, affordability, and integration into existing farm management systems are necessary. The integration of AI in rabbit husbandry can foster sustainable and humane practices, providing data-driven insights for better decision-making. This review highlights how AI innovations will revolutionize rabbit farming and lay the groundwork for broader applications in livestock management, contributing to the global expansion of precision farming.

## 1 Introduction

Rabbit farming plays a crucial role in commercial meat production and as a source of companionship for pet owners (1). Globally, rabbits are highly valued for their rapid reproductive rates, high feed conversion efficiency, and adaptability to various farming systems (1, 2). However, managing rabbit reproduction, health, and behavior presents significant challenges, especially in large-scale operations. These challenges are intensified by rabbits’ rapid reproductive cycles and relatively short lifespans, which demand continuous monitoring and prompt decision-making to safeguard their health, productivity, and welfare (3, 4). Traditional approaches to reproductive management, pregnancy detection, successful delivery, and health monitoring often rely on labor-intensive practices that require skilled personnel and are prone to human error, influenced by subjective judgment and environmental variability (2, 5). For instance, palpation and ultrasound, commonly employed for pregnancy confirmation, are non-invasive but require specialized training and equipment (6). In large-scale rabbit farms, reliance on manual methods can be overwhelming, leading to inefficiencies, delayed interventions, and potential compromises in animal welfare.

To address these limitations, there is a growing demand for efficient, accurate, and automated systems to manage rabbit reproduction and health (7). Artificial Intelligence (AI) has emerged as a promising solution, capable of streamlining and automating key aspects of rabbit husbandry. Technologies such as machine learning (ML), computer vision, and Internet of Things (IoT)-based systems enable continuous, non-invasive monitoring of health, fertility, and behavior, while providing real-time insights to support informed management decisions. AI has been demonstrated to be effective in detecting pregnancy, predicting labor, and providing postpartum care. For example, ML models can analyze physiological indicators, such as body temperature, heart rate, and activity levels, to anticipate the onset of pregnancy or labor, thereby minimizing complications (8). AI systems can also monitor does and their kits after parturition, detecting problems such as infections, insufficient milk production, or abnormal behavior, which allows timely intervention (9).

Moreover, AI-powered behavioral analysis tools are increasingly being applied to track essential activities, including feeding, drinking, grooming, and social interactions, thereby ensuring appropriate housing conditions and helping to reduce stress and aggression common issues in intensive farming environments (10). This review highlights the current applications of AI in rabbit farming, with a particular focus on pregnancy detection, labor prediction, postpartum care, health monitoring, and behavioral assessment. We also discuss the expanding body of research supporting AI integration into rabbit farm management systems and the specific techniques being developed for practical implementation.

This review also highlights the challenges associated with implementing AI in rabbit farming, including the need for standardized data collection methods, ethical concerns regarding continuous monitoring, and barriers to adoption in resource-constrained environments. Additionally, we discuss future directions for AI in rabbit husbandry, emphasizing the importance of innovation and the seamless integration of AI systems into sustainable, welfare-focused farming practices. The potential of AI to enhance productivity, reduce labor costs, and improve animal welfare in rabbit farming is substantial. As AI technology continues to evolve, it promises to transform traditional farming methods, leading to more efficient, humane, and sustainable approaches to animal husbandry. This article provides an overview of current advancements in AI for rabbit management, identifying key areas for future research and development, including rabbit pregnancy monitoring, health and behavior analysis, and environmental management.

## 2 AI advancements in rabbit management

**Figure 1:**
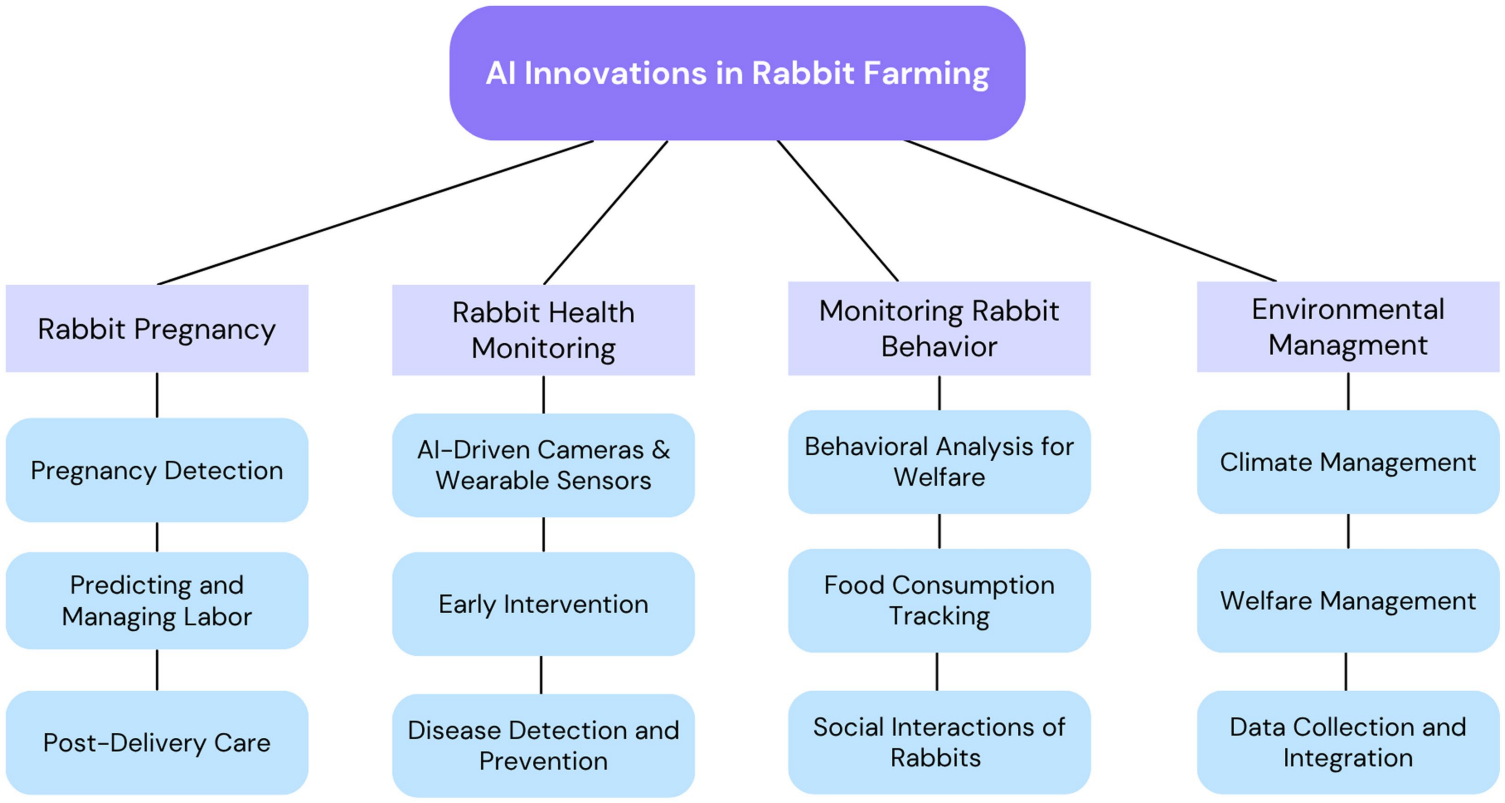
Taxonomy of artificial intelligence (AI) innovations in rabbit farming. This figure outlines the hierarchical classification of AI technologies and their core applications across rabbit production systems.

The integration of AI into animal agriculture has begun to redefine livestock management across multiple species, including rabbits. With the growing global emphasis on improving animal welfare, farm productivity, and environmental sustainability, AI presents a powerful set of tools to optimize rabbit farming. AI technologies offer advanced capabilities for automating routine tasks, predicting critical biological events, and facilitating data-driven decision-making. These innovations are particularly relevant such rabbit farming scales to meet increasing demands for meat, fur, and research animals, while also responding to stricter welfare standards. Figure 1 illustrates how AI is currently being applied across four primary domains in rabbit farming: pregnancy monitoring, health surveillance, behavioral tracking, and environmental management.

In rabbit reproduction, Artificial Intelligence (AI) has demonstrated strong potential to improve breeding efficiency through early and accurate pregnancy detection (7, 9). Traditional diagnostic methods, such as palpation, are labor-intensive, require skilled personnel, and may cause stress. AI-powered systems utilizing machine learning and computer vision can analyze external indicators, such as posture, activity levels, and subtle changes in temperature or respiration, providing a non-invasive and efficient alternative (5, 9, 10). These approaches can be further strengthened by integrating wearable biosensors for continuous monitoring of individual rabbits. Early pregnancy detection supports better planning of breeding programs, reduces unnecessary mating, and optimizes resource use. AI can also assist in monitoring late-gestation indicators and predicting labor, enabling timely interventions and improving kit survival during parturition (11, 12). Postpartum, AI-based smart monitoring systems track neonatal health by measuring temperature, nursing frequency, and movement within the nest, with automated alerts to identify abnormal conditions such as low activity or insufficient maternal care. Data from the postpartum phase can then refine predictive models, enhancing reproductive success and herd genetics over time (9, 10).

Beyond reproduction, AI is transforming health management into a new realm, not unlike rabbit farming. Camera- and biosensor-based systems can identify early signs of illness, including respiratory or digestive disorders, or indicators of pain and distress, often before they are visible to human observers (11, 13). Subtle changes in gait, posture, or feeding behavior may reveal emerging health problems. Continuous surveillance offers more consistent coverage than manual observation, as it combines behavioral and physiological data with environmental factors such as temperature, humidity, and air quality, thereby enabling context-aware diagnoses (2, 5). This proactive approach reduces disease outbreaks, improves welfare, enhances productivity, and lowers veterinary costs (9, 11).

Behavioral analysis is another frontier in precision rabbit farming. Rabbits are highly sensitive and social animals whose behaviors reflect their physical and psychological state (14). AI systems utilizing computer vision and motion analysis can track activity patterns, grooming behaviors, social interactions, and aggression. Deviations from normal behavior, such as reduced movement or altered feeding times, may precede clinical symptoms of disease. Moreover, AI can detect negative social interactions, such as chasing, biting, or dominance behaviors, supporting management decisions about group housing and enrichment (15, 16). These behavioral insights can also be applied to design tailored welfare programs that promote mental stimulation, reduce stress, and improve overall well-being (17).

**Figure 2:**
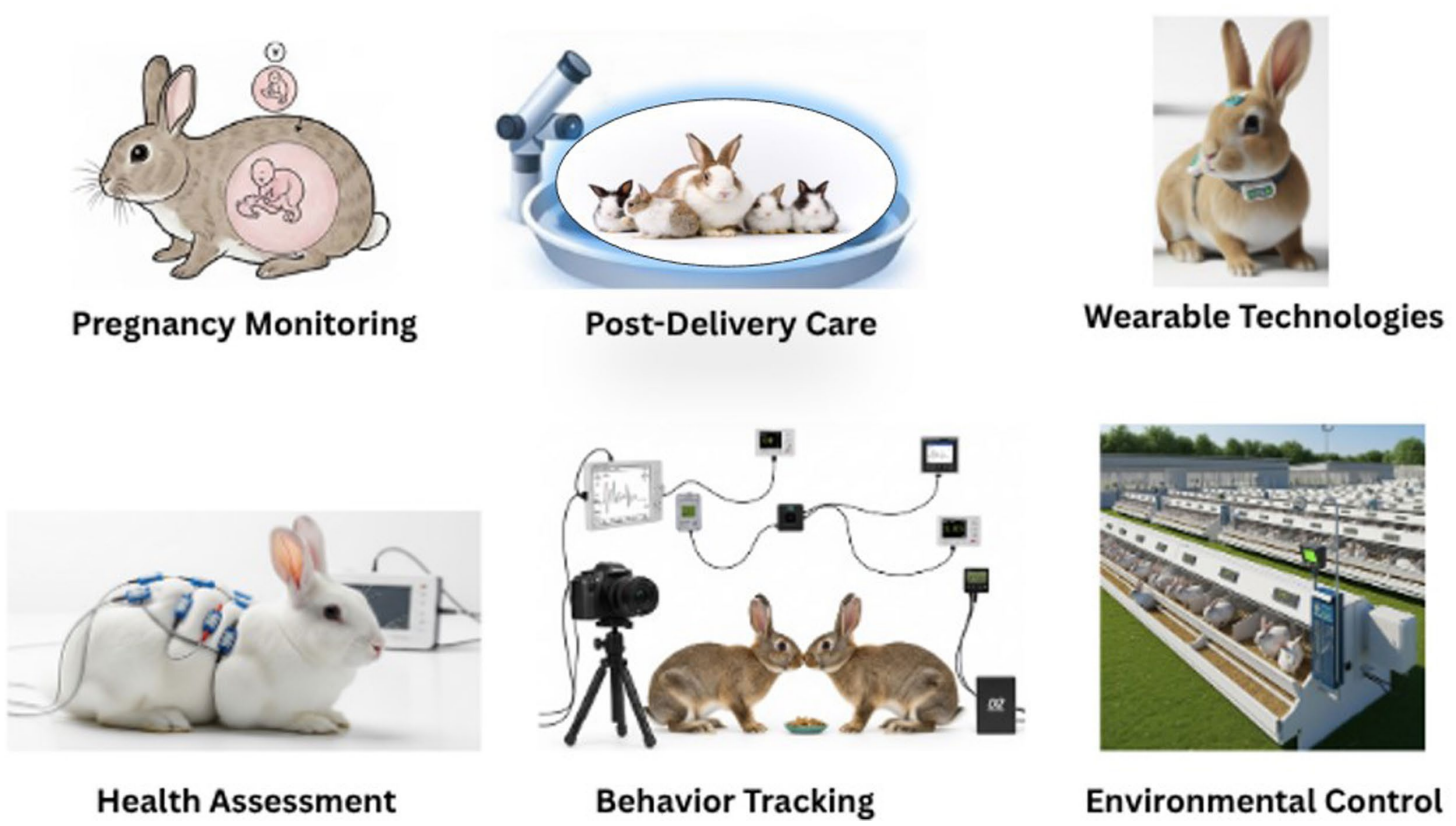
Overview of AI applications in rabbit farming. Key areas include sensor-based monitoring, wearable technologies, health assessment, behavior tracking, and environmental control.

Figure 2 provides a visual representation of the diverse applications of AI in rabbit management. It includes six key components: pregnancy monitoring, post-delivery care, wearable technologies, health assessment, behavioral tracking, and environmental control. Each of these components contributes to a data-rich, responsive farming system that enables early detection of issues, real-time intervention, and continuous welfare optimization. Wearable technologies are revolutionizing the collection of data at the individual animal level. Lightweight devices can measure physiological parameters, such as body temperature, heart rate, and movement, and transmit this data to AI platforms for real-time analysis. This granular data supports individual health profiling, enabling more precise and personalized care.

In addition to monitoring animals, AI plays a crucial role in managing the farming environment. Environmental conditions, including temperature, humidity, air flow, and light exposure, have a direct impact on rabbit health, productivity, and welfare. AI-integrated climate control systems can automatically adjust these parameters in response to real-time sensor inputs, maintaining stable and optimal living conditions. Moreover, AI can utilize predictive analytics to forecast environmental challenges, such as heatwaves or cold spells, based on external weather data, enabling farmers to prepare in advance (9, 17). This predictive capability not only protects animal welfare but also improves energy efficiency and reduces operational costs by avoiding unnecessary overuse of heating or cooling systems.

Another promising avenue is the integration of AI data across all these domains into centralized farm management platforms. Such platforms can provide comprehensive dashboards that track reproductive performance, health status, behavioral trends, and environmental metrics in real time. Decision-support tools powered by AI can offer actionable insights, suggest interventions, and even automate routine responses. For instance, if an AI system detects a drop in activity coupled with high ambient temperatures, it may automatically activate cooling systems while alerting farm personnel to check for signs of heat stress.

In summary, the incorporation of AI into rabbit management is transforming the way farms operate, moving from reactive, labor-intensive practices to proactive, data-driven systems. By integrating technologies that support pregnancy detection, health monitoring, behavioral analysis, and environmental control, AI enables a holistic approach to animal welfare and farm efficiency. As illustrated in Figures 1, 2, the synergy between these AI applications not only enhances productivity but also aligns with evolving ethical and sustainability standards in modern animal agriculture.

## 3 AI in monitoring rabbit pregnancy

**Figure 3:**
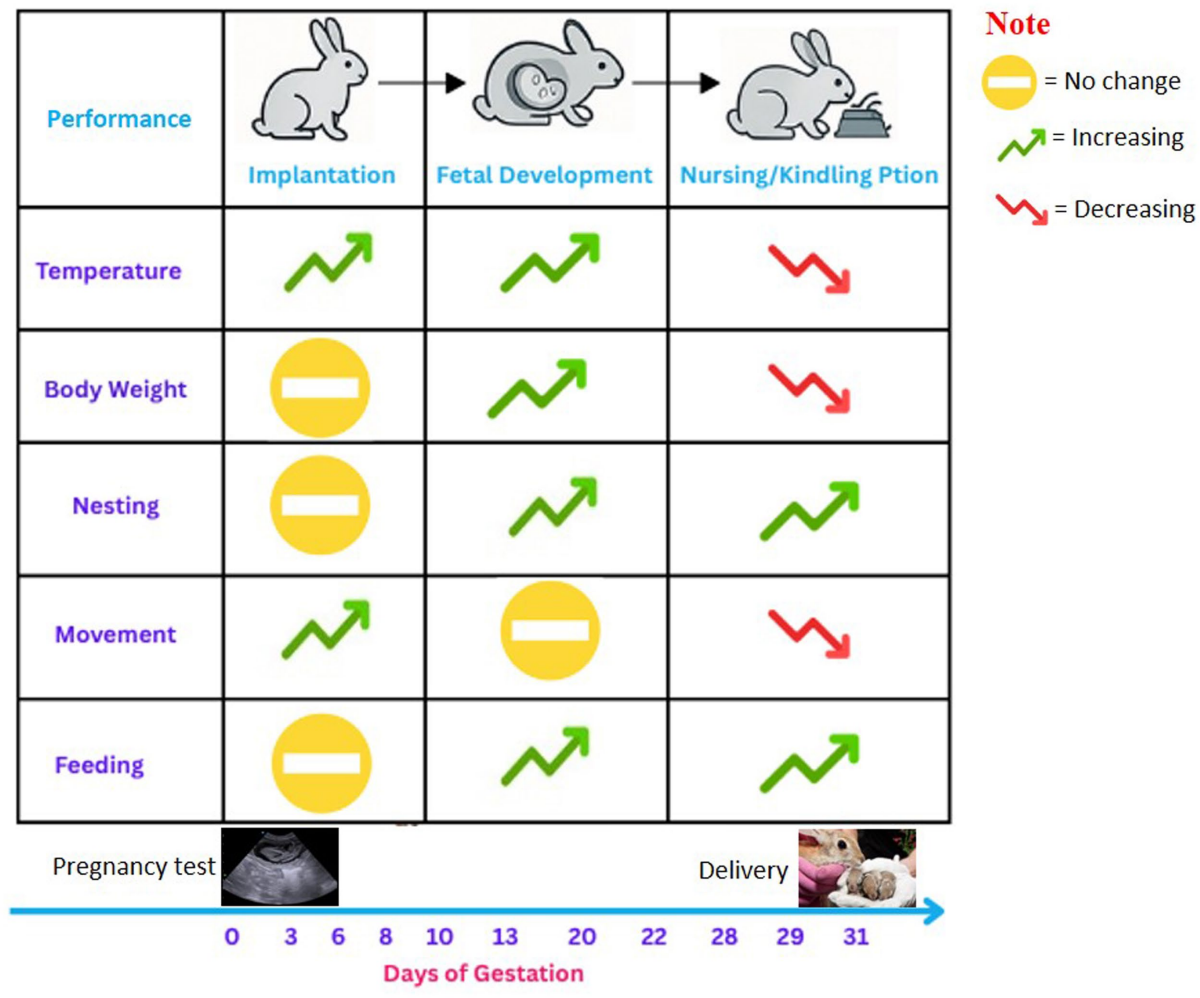
AI applications for pregnancy monitoring in rabbits. The figure illustrates how AI-based techniques contribute to early detection of estrus and pregnancy status.

Figure 3 illustrates an AI-based system for monitoring rabbit pregnancy, tracking physiological and behavioral changes across three major gestational phases: implantation (Days 0–6), fetal development (Days 8–22), and nursing/kindling preparation (Days 28–31). Scientific literature (6, 7, 12, 15) supports the observed trends shown in the figure. During implantation, gradual increases in core body temperature and movement are consistent with early hormonal and metabolic shifts. Nesting and feeding behaviors remain minimal in this phase. As pregnancy progresses into the fetal development stage, steady rises in temperature, body weight, feeding activity, and nesting behavior are observed, corresponding to increased fetal growth and maternal resource demands. Movement becomes more stable during this period, as supported by studies utilizing AI and accelerometers to monitor behavioral patterns in rabbits. In the final phase, nursing and kindling preparation, marked physiological and behavioral shifts occur. Research shows a characteristic decline in maternal body temperature just before kindling, followed by a sharp rise at parturition. Body weight and feeding behavior may also decrease due to hormonal changes and the physical burden of late gestation (6, 15). Nesting behavior, regulated by hormonal cues such as estrogen and prolactin, intensifies significantly during this phase, with does engaging in fur-pulling and active nest-building in preparation for kindling. Simultaneously, movement typically decreases, as the doe conserves energy and remains close to the nesting site. By analyzing various biological markers and environmental factors, AI may predict key milestones in the pregnancy, such as the expected time of birth or any potential complications. This predictive capability helps farmers and veterinarians to plan and prepare for each stage of pregnancy, enhancing the efficiency and success rate of breeding programs. Integrating AI in pregnancy monitoring may offer a more precise and data-driven approach to rabbit reproduction, ultimately improving overall care and management practices.

### 3.1 Pregnancy detection

Accurately detecting pregnancy in rabbits is crucial for optimizing breeding cycles, reducing unnecessary mating, and improving herd management efficiency (12). Traditional methods, such as manual palpation and ultrasound, are widely used for pregnancy detection but are often stressful and semi-invasive, require skilled personnel, and may affect animal welfare and productivity (6, 12).

Artificial intelligence (AI) technologies, including machine learning, computer vision, and sensor integration, have emerged as promising tools to enhance the accuracy of pregnancy detection while minimizing labor demands and animal stress (14). One of the most promising advancements in this area is the use of non-invasive sensors to monitor physiological changes in rabbits during pregnancy. For instance, thermal infrared imaging can detect subtle temperature fluctuations associated with hormonal changes in pregnant rabbits. AI algorithms can analyze these thermal patterns to predict pregnancy with high accuracy (12, 16). Reported accuracy rates for AI-based thermal imaging systems vary depending on model, dataset size, and environmental conditions but typically range from 80 to 95%, which is higher than traditional palpation or manual inspection methods (18). Continuous monitoring of body temperature and other physiological parameters enables AI systems to provide ongoing assessments of pregnancy status, minimizing the need for physical handling, facilitating early intervention, and enhancing overall reproductive management efficiency. Wearable biosensors are another AI-driven solution to gain traction in the monitoring of rabbit pregnancies. These sensors, which attach directly to the animals, can continuously track physiological parameters such as heart rate, temperature, and movement. The real-time data generated is analyzed by machine learning models to predict pregnancy, labor onset, and overall health status. For instance, a recent study by Yuan et al. (19) introduced a wearable device that uses photoelectric sensors to assess physiological signals associated with pregnancy. This approach offers continuous, non-invasive monitoring, minimizes human error, and improves decision-making in farm management (12, 18, 19).

AI-based image recognition systems have also been developed to detect visual changes in rabbits that may indicate pregnancy. These systems monitor physical characteristics such as abdominal size and shape, which are often early signs of pregnancy. AI-powered computer vision can analyze these changes with high precision, alerting farmers to the presence of pregnancy and enabling earlier interventions and optimized breeding cycle management (20). Furthermore, these technologies enable long-term visual monitoring without physical contact, thereby enhancing both animal welfare and operational efficiency.

Table 1 summarizes recent advancements in AI-based techniques for early pregnancy detection in rabbits. Multiple AI models, including Support Vector Machine (SVM), Partial Least Squares Discriminant Analysis (PLS-DA), K-Nearest Neighbors (KNN), Naive Bayes, and Convolutional Neural Networks (CNN), have been applied to diverse input datasets such as optical reflectance, Vis–NIR spectroscopy, and image-based features. Among rabbit studies, SVM models using optical data from wearable or handheld sensors have demonstrated high accuracy (up to 86.63 and 83.94%, respectively) in detecting pregnancy as early as 14 days post-insemination.

**Table 1:** Early detection of estrus and pregnancy in rabbits.

AI technique used	Input data	Objectives/Goal	Animal model	References
Medium gaussian support vector machine (SVM)	Optical data collected by RAPID device using 8 sensor modules with LEDs emitting 660 nm, 850 nm, and 940 nm; Reflectance data from abdominal gestational sac tissue; Multiple data points from does of varying age and hair density	Detection of early pregnancy with up to 86.63% accuracy on day 14 post-insemination	Rabbit	(37)
Partial least squares discriminant analysis (PLS-DA), support vector machine (SVM), K-nearest neighbors (KNN), and Naive Bayes	Visible–Near Infrared (Vis–NIR) spectra ranging from 490 to 960 nm were collected at various source-detector distances (SD-5, SD-10, and SD-15). The spectral data were preprocessed using techniques such as Median Filtering (MF), Standard Normal Variate (SNV), and Min–Max normalization. Feature selection was performed using Successive Projections Algorithm (SPA) and Competitive Adaptive Reweighted Sampling (CARS). The classification was based on pregnancy status, distinguishing between pregnant rabbits (PRs) and non-pregnant rabbits (NPRs).	Detection of early pregnancy with highest prediction accuracy up to 90.69% using SPA-SVM	Rabbit	(18)
Support vector machine (SVM), Partial least squares discriminant analysis (PLS-DA)	Optical reflectance data from wearable device using 3 LEDs (660, 850, 930 nm) and 2 Si-based photodiodes; 419 rabbit samples with varying hair density (dense, sparse, hair removal); Reflected light intensity from abdominal tissue	Detection of pregnancy status in rabbits; Best performance with hair-removed rabbits using SVM: 83.94% accuracy, 83.67% recall, 81.19% precision, 82.41% F-score	Rabbit	(19)

Despite these advancements, challenges remain in system integration and widespread adoption. Implementing AI solutions requires investment in hardware and software, which may be a barrier for small-scale farms. Additionally, data collection and integration must be standardized to ensure consistent accuracy across different farming environments. Nonetheless, the potential of AI to revolutionize pregnancy detection in rabbits and improve herd management practices is significant. As AI continues to evolve, it is expected to play a crucial role in making rabbit farming more sustainable, efficient, and welfare-friendly (12, 14).

### 3.2 Estimating gestation period

Due to variation in gestation length in rabbit breeds, accurate prediction of the gestation period in rabbits is essential for optimizing breeding cycles, reducing risks associated with premature or delayed births, and improving overall farm management (Figure 3). While the typical gestation period in rabbits is approximately 31 days, variations can occur depending on the breed, age, health status, and environmental factors (6). Traditionally, gestation predictions have relied on historical experience and general guidelines, which are often imprecise. The introduction of AI and machine learning technologies now allows for more accurate predictions of the gestation period by analyzing multiple data points, including physiological indicators and environmental conditions. Machine learning models can be trained on historical breeding data to identify patterns and correlations, enabling more precise prediction of individual delivery dates (8). This approach provides a clear link between AI-based data analysis and improved reproductive management, bridging the gap between traditional estimations and real-time, individualized predictions. For instance, AI systems can analyze data from previous pregnancies to understand the typical gestation length of specific rabbits or breeds, thereby providing a more personalized and accurate prediction. Additionally, factors such as the rabbit’s age, body condition, and reproductive history are taken into account by machine learning algorithms to fine-tune predictions further and ensure the accurate estimation of the expected delivery date (7). By incorporating such data, farmers can make more informed preparations for birthing, thereby improving the management of both the doe and her kits.

Environmental factors, including temperature, humidity, and stress, also play a critical role in the duration of gestation (9). AI systems can integrate these variables using data from weather forecasts or environmental sensors deployed on the farm. Research has shown that fluctuations in temperature and environmental stressors can influence the gestation period in rabbits (20). AI-powered models can process these external factors alongside physiological data to offer a more holistic and accurate prediction of the gestation period. By accounting for internal and external factors, AI systems provide a comprehensive tool for enhancing farm management and mitigating uncertainties associated with labor allocation.

Moreover, integrating wearable devices and sensors into AI systems allows real-time monitoring of the doe’s physiological signals, such as body temperature, heart rate, and activity levels. These devices can track subtle changes that may indicate impending labor, further enhancing the accuracy of gestation predictions (19). The continuous data flow from such sensors, combined with advanced machine learning algorithms, can provide real-time alerts about potential complications or deviations from the expected gestation period. This allows farmers to take timely actions, such as adjusting environmental conditions or providing additional care, to mitigate risks to the doe and her offspring. As AI systems continue to evolve, their ability to predict and monitor gestation periods will likely become more refined, improving the efficiency of rabbit farming practices and enhancing animal welfare (6, 18).

## 4 AI in monitoring rabbit delivery (parturition)

Table 2 highlights the diverse applications of AI in overseeing rabbit deliveries, offering essential support throughout the entire birthing process. One significant application is AI’s ability to predict the onset of labor, allowing for better timing and preparation. By analyzing various data points, such as temperature fluctuations, behavioral changes, and physiological signals, AI can forecast when a doe is likely to begin labor. This early prediction enables caregivers to make the necessary preparations, thereby reducing the risk of complications during delivery (16). It also provides a critical window for veterinarians or caretakers to intervene, ensuring a safer and smoother birthing experience for the mother and her kits.

**Table 2:** Applications of AI in rabbit parturition and post-delivery care.

AI application	Description	References
Labor onset forecasting	AI predicts labor onset by analyzing temperature, behavioral changes, and physiological data, allowing timely prep.	(11, 18, 19)
Uterine contraction monitoring	Tracks contraction frequency, intensity, and regularity to assess labor progress and detect complications early.	(9, 12)
Behavioral pattern recognition	Detects nesting, restlessness, and posture changes indicating imminent parturition with high accuracy.	(12, 17–19)
Environmental integration	Incorporates environmental data (temperature, humidity, stress) for holistic labor management and smoother delivery.	(20)
Real-time health monitoring	Continuously monitors doe’s vital signs (temperature, heart rate, respiration) during labor and post-delivery.	(12, 16, 17)
Decision-support systems	Provides automated alerts and actionable recommendations to reduce risks of dystocia and improve birthing outcomes.	(7, 12)
Maternal recovery monitoring	Tracks post-delivery recovery signs, identifying infections or uterine complications early to improve health outcomes.	(12, 16)
Milk production and nursing analysis	Monitors milk production and nursing behavior to ensure kits receive adequate nutrition, alerting for supplementation.	(6, 7)
Kit activity and vitality assessment	Analyzes kit movements and feeding behavior to detect distress or lethargy, ensuring timely intervention.	(17, 18)
Maternal behavior surveillance	Evaluates maternal care behaviors like grooming and nursing, alerting to deviations to safeguard the kit’s survival.	(9, 10)
Precision livestock management	Supports resource-efficient, targeted care for individual animals, reducing labor costs and improving welfare.	(10, 22)

Furthermore, Table 2 illustrates how AI assists in managing both labor and post-delivery care. During labor, AI tools can monitor contraction progress, track the positioning of the kits, and assess the overall health of the doe in real-time. If any irregularities or delays are detected, the system can alert caregivers to take timely action (12). After delivery, AI continues to play a vital role by monitoring the mother’s recovery and the well-being of the newborns. It can identify potential signs of infection or distress, suggest appropriate feeding plans, and ensure that both the doe and her kits are receiving proper care (17, 19). By integrating AI into these processes, the management of rabbit deliveries becomes more efficient, ultimately leading to improved outcomes for both the doe and her offspring.

### 4.1 Predicting and managing labor

The labor process in rabbits can be challenging to predict, and complications such as dystocia (difficult labor) or stillbirth can occur. AI can be vital in predicting and managing parturition by analyzing real-time physiological and behavioral data. The labor process in rabbits, commonly known as parturition, is a critical phase in rabbit husbandry. However, it is often difficult to predict, and complications such as dystocia or stillbirth can occur, which pose significant risks to both the doe (female rabbit) and her kits. Accurate prediction and management of labor are crucial to improving outcomes and ensuring the health of the animals (17). In recent years, AI has emerged as a powerful tool for monitoring and predicting labor by analyzing a range of real-time physiological and behavioral data. AI algorithms can effectively process data from wearable sensors, such as temperature sensors, accelerometers, and heart rate monitors, to detect subtle changes in the doe’s body that indicate the onset of labor (18). These sensors continuously track vital signs, allowing AI systems to detect early warning signals and provide real-time insights into the progress of parturition.

Wearable sensors are among the most innovative tools in AI-driven labor monitoring. For example, accelerometers can monitor changes in the doe’s activity level, while temperature sensors detect slight increases in body temperature that typically occur as labor approaches (19). AI models, when fed with historical data and real-time inputs from these sensors, can identify patterns of behavior and physiological changes associated with the early stages of labor. When the system detects patterns of restlessness, such as nesting behavior, increased movement, or changes in posture, it can predict with high accuracy that parturition is imminent. These early warnings can alert farmers or veterinarians, allowing them to intervene if necessary, minimizing the risks of complications and ensuring better management of the birthing process (17–19).

In addition to monitoring behavioral changes, AI can also be employed to track uterine contractions during labor. Contraction monitoring is crucial for assessing the progress of labor and identifying potential complications, such as uterine inertia, which can impede the birthing process. By analyzing the frequency, intensity, and regularity of contractions, AI systems can provide an accurate estimate of the time remaining before delivery (9). AI-based tools can process this data in real-time, providing farmers with valuable information that helps them anticipate when labor is likely to occur and whether medical intervention may be necessary. Such predictive capabilities can greatly reduce the occurrence of stillbirths or injuries to the doe and her kits, improving both productivity and animal welfare on the farm (6, 12).

Furthermore, AI’s ability to analyze vast amounts of data from multiple sensors and sources allows for the integration of various factors that can influence labor. For example, environmental conditions, such as temperature, humidity, and stress levels, can also impact the timing and difficulty of labor (20). AI models can incorporate these external factors by processing data from environmental sensors in conjunction with physiological data from the doe. By integrating a broader range of variables, AI can offer a more holistic understanding of the factors influencing parturition, enabling farmers to optimize conditions for a smoother and less stressful delivery. Integrating AI-driven labor prediction with environmental monitoring is particularly valuable in large-scale commercial rabbit farms where managing labor manually can be challenging.

AI-based labor prediction tools are also evolving to include automated alerts and decision-support systems that assist in decision-making during labor. For instance, AI systems can generate real-time alerts for farmers when labor complications, such as the risk of dystocia, are detected. These alerts can be based on real-time analysis of the doe’s physiological state, behavior, and uterine contractions. AI tools can also suggest appropriate actions, such as providing additional care, increasing observation, or preparing veterinary intervention if needed (7). These advanced AI capabilities enable more proactive management of parturition, ensuring that any complications are addressed promptly to prevent delays or mortality during labor.

Finally, integrating AI systems into the management of rabbit parturition holds the potential to transform breeding practices in the long term. AI can not only assist in real-time labor monitoring but also contribute to the broader breeding management strategy by identifying factors that improve reproductive success. AI technologies have increasingly been applied in rabbit reproduction to improve accuracy and efficiency at every stage, from pregnancy detection to labor management and postpartum care. Accurate detection of pregnancy is crucial for optimizing breeding cycles, reducing unnecessary mating, and improving herd management efficiency (19, 20). Traditional methods, such as manual palpation and ultrasound, are often stressful, require skilled personnel, and may affect animal welfare and productivity (6, 18). Non-invasive AI-based approaches, including Vis–NIR spatially resolved spectroscopy and wearable photoelectric sensors, allow continuous monitoring of physiological changes associated with pregnancy, such as subtle temperature fluctuations, movement patterns, and other biometric signals (18, 19). These AI systems can analyze complex datasets in real time, providing pregnancy predictions with higher accuracy than conventional methods, while minimizing handling stress.

Beyond pregnancy detection, AI can monitor uterine contractions and other physiological indicators critical for understanding labor progress. By analyzing the frequency, intensity, and patterns of contractions, AI algorithms can predict the timing of parturition, enabling timely interventions to support both the doe and her kits (18, 19). The integration of advanced imaging techniques, such as *in vivo* ultrasound and biomicroscopy, further enhances AI’s ability to visualize uterine and follicular activity, thereby improving the precision of labor monitoring (20). Continuous observation also enables the early detection of complications, such as prolonged labor or dystocia, facilitating rapid corrective actions and reducing maternal mortality (21).

Postpartum, AI systems continue to play a pivotal role in neonatal care. Smart monitoring tools can track kit activity, nursing frequency, body temperature, and movement within the nest, alerting farmers to potential issues such as low maternal care or abnormal kit behavior. The data collected during the postpartum period can be used to refine AI models over time, improving predictive accuracy and supporting long-term reproductive success. Overall, these AI-driven approaches not only enhance animal welfare and productivity but also allow targeted resource allocation, reduce routine labor demands, and support more informed decision-making in large-scale rabbit farming (18–21).

### 4.2 Post-delivery care

Post-parturition care is another area where AI can improve rabbit health and welfare. After delivery, both the doe and her kits require careful monitoring to ensure they are recovering well and receiving proper care. AI technologies, such as wearable health trackers and video monitoring systems, can be used to assess the health of both the mother and her offspring (12, 16). Likewise, post-parturition care is an essential aspect of managing rabbit health and welfare, as both the doe and her kits require consistent monitoring after birth. AI technologies offer significant improvements in the way farmers track the health of the doe and her offspring. Wearable health trackers and AI-based video monitoring systems are increasingly used to assess vital signs such as body temperature, heart rate, and respiratory rate, which are critical for detecting postpartum complications like infections or uterine issues (12). These systems provide continuous, real-time data analysis, enabling farmers to detect health issues early, thereby reducing the risk of serious complications and improving recovery outcomes.

AI can also help monitor the doe’s milk production, a crucial factor for the survival of the kits. Ensuring adequate milk production is essential for the kits’ nutrition and growth, as insufficient nursing can lead to malnutrition. Through the use of sensors and video analysis, AI systems can track the doe’s nursing behavior, confirming whether the kits are nursing properly and receiving adequate nourishment. If the system detects that kits are not nursing or that milk production is insufficient, AI algorithms can alert the farmer to take targeted interventions. These may include providing supplemental milk or formula feeding, warming or adjusting the nest to improve kit comfort, performing health checks on both the doe and kits, or temporarily fostering kits to another lactating doe to ensure adequate nutrition (6, 7). By enabling timely and precise interventions, AI systems such as computer vision platforms for behavior tracking, wearable sensor networks for physiological monitoring, and IoT-enabled smart nest monitoring devices can alert farmers when kits are not nursing or when milk production is insufficient (6, 7). These systems enable timely interventions, including supplemental feeding, nest adjustments, or health checks, ensuring the welfare of both the doe and her offspring. Additionally, AI systems can be used to monitor the activity levels of the kits themselves, providing crucial insights into their overall health and development. Monitoring kit behavior through video footage or wearable sensors can reveal important information about feeding habits, physical activity, and overall vitality (17). By analyzing these behaviors, AI algorithms can detect signs of distress, lethargy, or other health issues that could hinder the kits’ growth. If abnormalities are detected, the AI system can alert the farmer to take immediate action, ensuring that the kits receive the care they need to thrive (18). This type of early intervention can significantly improve survival rates in kits, especially in large-scale rabbit farms where manual monitoring may be less feasible.

Moreover, AI can enhance the monitoring of maternal behaviors, such as nursing and grooming, which are vital for the kits’ survival in the early post-delivery period. AI systems can track these behaviors through video analysis, identifying issues such as reduced grooming or failure to nurse that could compromise the kits’ development. If the AI system detects deviations from normal patterns, it can send real-time alerts to the farmer, prompting immediate intervention (9, 10). By integrating these AI-based monitoring systems into farm management practices, farmers can ensure the optimal welfare of the doe and her kits, addressing potential issues before they become critical.

Furthermore, the integration of AI-based systems in post-delivery care aligns with the growing trend in precision agriculture and livestock management. AI technologies can support the efficient use of resources by enabling farmers to provide targeted care based on individual animal needs rather than applying a one-size-fits-all approach. These systems also contribute to reducing labor costs, as they automate the monitoring process and provide valuable insights without requiring constant human intervention. As AI and sensor technologies continue to advance, they are expected to play even more prominent role in improving animal welfare and productivity across rabbit farms (10, 22). AI-based systems can also monitor kit activity levels, which can indicate whether they are thriving post-birth.

## 5 AI in rabbit health monitoring

Table 3 demonstrates the various applications of AI in monitoring rabbit health, emphasizing its role in disease detection, prevention, and ongoing health tracking through wearable devices. One key application is AI’s capability to identify early signs of illness by analyzing data from multiple sources, such as changes in behavior, physiological indicators, and environmental conditions (12, 16). Through advanced machine learning techniques, AI can detect patterns and anomalies, enabling early intervention before the condition escalates. This proactive approach to disease detection helps minimize health risks and contributes to the overall well-being of the rabbits.

**Table 3:** AI techniques for disease prediction and health monitoring in rabbits.

AI technique used	Input data	Target disease(s)	Goal/Objectives	Animal model	Reference
Expert system using forward chaining and certainty factor (CF) method	User-entered symptom data (e.g., loss of appetite, weight loss, lethargy, ear redness); Expert-assigned CF values per symptom-disease association	Scabies, Gastroenteritis, Otitis, Conjunctivitis, Hypocalcemia, Pneumonia	Web-based diagnostic tool for predicting rabbit diseases and providing treatment recommendations; Generates CF-based confidence levels (e.g., 99.99% for Scabies)	Rabbit	(33)
Hybrid deep neural network: convolutional neural networks (CNNs) and gated recurrent units (GRUs)	Heart sound (HS) signals from New Zealand rabbits at four time points during repeated exhaustive swimming; Processed using STFT, double threshold segmentation, and FFT	Exercise-induced sudden cardiac death	Classify and predict rabbits’ risk of sudden death due to excessive exercise with 89.57% accuracy, 89.38% sensitivity, and 92.20% specificity	Rabbit	(38)
Random forest classifier (RFC)	Severity scores of eye damage (cornea, conjunctiva, and eyelids), including erosion, cloudiness, and abnormal secretions, assessed at 48 h, 72 h, 96 h, and 1 week after sulfur mustard (SM) vapor exposure; 166 rabbit eyes evaluated	Sulfur mustard-induced ocular injury (prediction of late corneal neovascularization)	Predict development of late ocular pathology (corneal NV) 4 weeks post-exposure; 73% overall accuracy, 75% PPV, 59% NPV; most predictive features were conjunctival secretion and corneal opacity at 1w, and corneal erosion at 72 h	New Zealand White Rabbit	(40)
Naïve Bayes classifier with certainty factor (CF) method	User-input symptoms (e.g., alopecia, crust, loss of appetite, mucous feces); CF values from users and experts; 65 to 160 learning records	Scabies, Malocclusion, Earmites, Furmites, Diarrhea, Flu, Worms, Coccidiosis, Eye Inflammation, Hairball	Android-based expert system to classify rabbit diseases with increasing accuracy (53% with 65 data; 73% with 160 data); Provides diagnosis and treatment advice with confidence score	Rabbit	(36)
Biological neural network with EYOLOv5 (Enhanced YOLOv5) and Weighted DIOU-NMS	Sensor network data (temperature, humidity, ammonia, pH, light); Visual monitoring via rabbit detection from camera input; Environmental threshold violations	Environment-induced diseases (respiratory, skin, low immunity, reproductive issues); Monitoring failure alerts	Real-time detection and prediction of farm environmental faults and rabbit presence; Intelligent deployment of wireless sensors for optimized coverage (up to 99.8%)	Rabbit	(39)

CF: Certainty Factor; HS: Heart Sound; STFT: Short-Time Fourier Transform; FFT: Fast Fourier Transform; NV: Neovascularization; PPV: Positive Predictive Value; NPV: Negative Predictive Value; SM: Sulfur Mustard.

**Figure 4:**
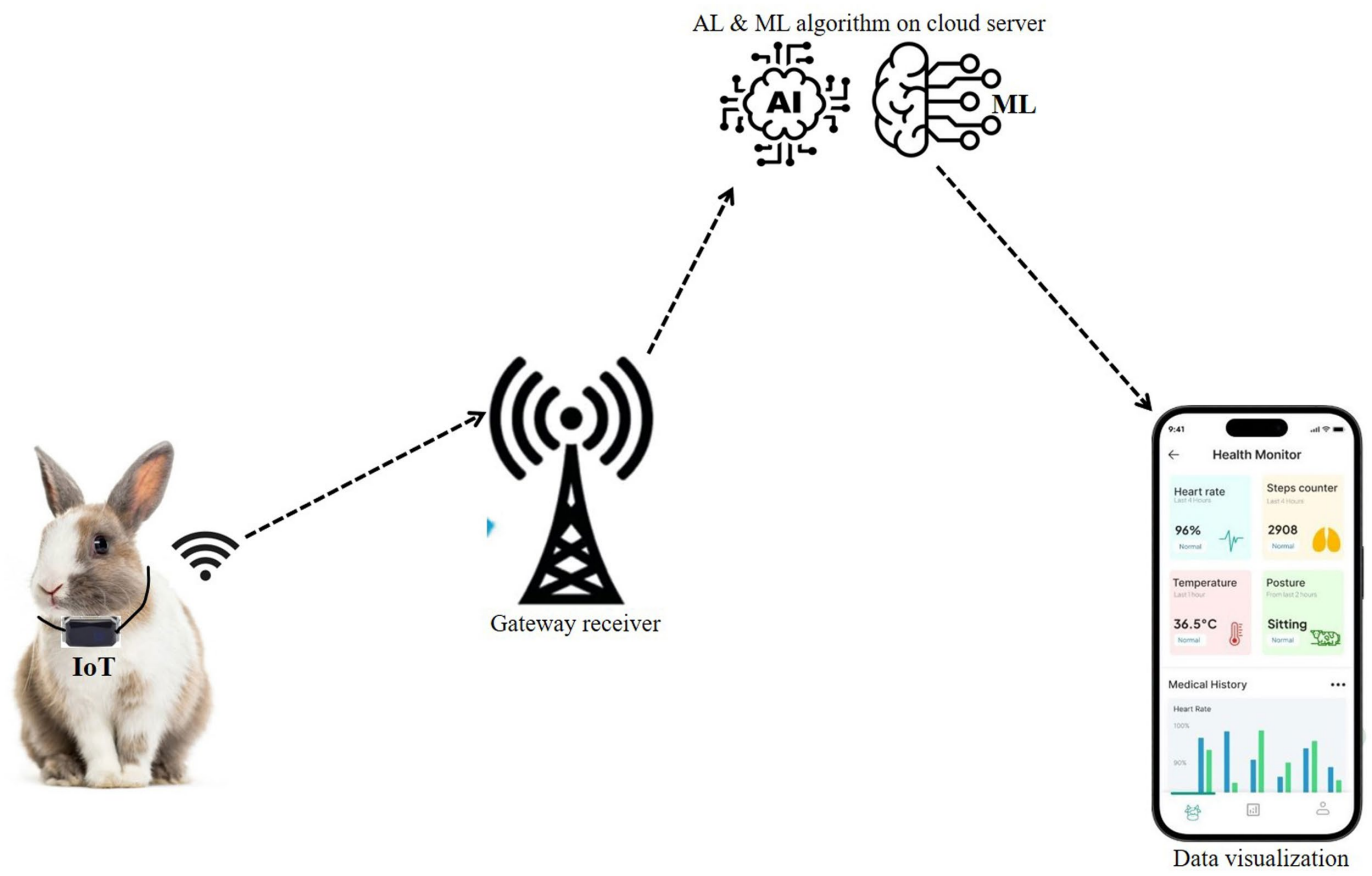
AI in rabbit health monitoring and disease prevention. This figure highlights AI tools used for early disease diagnosis, health tracking, and preventative care.

Additionally, Figure 4 showcases how wearable devices are integrated with AI to provide continuous health monitoring for rabbits. Wearables, such as collars or sensors, equipped with AI-powered algorithms, monitor vital signs like heart rate, body temperature, and activity levels in real-time. This constant stream of health data allows caregivers to identify any irregularities, ensuring prompt attention when needed. By combining AI-driven disease detection with real-time health monitoring through wearable devices, caregivers can significantly enhance the accuracy and efficiency of health management, leading to improved outcomes and increased well-being for the rabbits.

### 5.1 Disease detection and prevention

Early disease detection is essential for maintaining rabbit health and preventing outbreaks of potentially fatal diseases such as Myxomatosis, rabbit hemorrhagic disease (RHD), and various respiratory infections. Table 3 highlights various AI applications used to predict diseases and monitor health in rabbits. These AI models analyze data such as symptoms, heart sounds, environmental conditions, and eye or skin conditions to detect common illnesses like scabies, diarrhea, pneumonia, and stress-related heart issues. Systems include expert-based diagnostic tools, neural networks, and machine learning classifiers, offering early warnings and treatment suggestions. Some models also use real-time data from sensors and cameras to track environmental risks and rabbit behavior on farms. Overall, these technologies help improve rabbit health management through timely, accurate, and automated monitoring.

AI-based systems, particularly those leveraging computer vision and machine learning algorithms, offer significant potential for early detection of health issues in rabbits by continuously monitoring both physical and behavioral changes. These technologies can identify subtle signs of illness that might otherwise go unnoticed, allowing timely interventions that can prevent disease spread and protect entire herds (23).

Computer vision platforms can detect visible indicators such as changes in coat condition, eye appearance, or posture, which often signal the onset of illness. Simultaneously, AI algorithms can track behavioral alterations, including reduced activity, abnormal movements, or lethargy, alerting farmers to potential health concerns. For example, machine learning models can recognize patterns associated with discomfort or early-stage infections, enabling real-time alerts and rapid response to emerging problems (7, 23).

Beyond visual and behavioral monitoring, AI-based disease detection can integrate additional data sources, including health records, environmental sensors, and weather information. Machine learning models can analyze correlations between environmental variables such as temperature, humidity, and disease incidence to predict outbreaks before they occur (21, 23). For instance, certain pathogens may proliferate under specific seasonal or climatic conditions, and AI algorithms can identify these risk patterns. By providing early warnings, such systems empower farmers to implement preventive measures, thereby reducing the likelihood of widespread disease and improving overall herd health management.

Furthermore, AI technology can streamline diagnostic processes. In recent years, diagnostic tools such as the Luminex x-TAG assay have been developed to rapidly detect multiple rabbit pathogens, offering an example of AI’s potential in identifying the presence of disease-causing agents (21). These systems can be integrated into monitoring frameworks to provide farmers with more accurate and efficient diagnostic capabilities. By combining data from health records with real-time sensor data and predictive algorithms, AI systems can detect potential health threats and recommend appropriate treatment options much more efficiently than traditional methods (13).

Integrating machine learning algorithms into disease prevention can also help optimize farm management practices. AI models can process large datasets that include historical health trends, environmental variables, and current health monitoring data. This allows for a comprehensive understanding of the health dynamics within a farm, helping farmers to anticipate and mitigate disease risks proactively. For example, patterns emerging from data collected over time may indicate an increase in respiratory problems in a certain farm area, enabling farmers to address the issue before it escalates into a widespread problem (17). This data-driven approach not only enhances disease detection but also improves the overall health and welfare of the rabbit population.

Finally, AI can contribute to more sustainable and cost-effective farm management by minimizing the need for frequent manual inspections. AI systems equipped with sensors and machine learning models continuously monitor rabbit health and behavior, generating alerts only when an intervention is required (9, 10). This targeted approach ensures that labor and resources are used efficiently, allowing farmers to focus on complex management tasks while maintaining high standards of animal care. By integrating multiple monitoring technologies into a unified AI framework, farmers gain comprehensive health surveillance, improve decision-making, and enhance disease prevention and overall herd management.

### 5.2 Continuous health monitoring with wearables

Wearable sensors are increasingly recognized as crucial tools for continuous health monitoring in rabbits, providing farmers with real-time insights into the well-being of their animals. These devices, which can be attached as collars, implants, or tags, collect a variety of physiological data, including heart rate, body temperature, respiratory rate, and movement patterns (24). The data collected is then transmitted to AI models for analysis, which helps to detect early signs of illness or distress, even before physical symptoms become visible. This continuous monitoring allows for early intervention, potentially preventing disease progression and ensuring the overall health of the rabbits (13).

Smart collars, tags, or even subcutaneous implants can provide a non-invasive solution for tracking the health of rabbits around the clock. These devices provide accurate and real-time data on the animal’s vital signs, and AI algorithms can quickly detect any abnormalities. For instance, if a rabbit’s body temperature rises significantly, indicating fever, or its heart rate becomes irregular, signaling stress or potential health issues, AI systems can alert the farmer immediately (16, 20). Early detection is particularly critical in fast-paced farm environments, where traditional observation may not be sufficient or timely enough. By promptly identifying signs of illness such as infections, stress, or discomfort, these systems can prevent the spread of disease and ensure that the affected rabbit receives immediate care.

Additionally, wearable sensors can monitor behavioral changes, providing a more comprehensive understanding of a rabbit’s health. These sensors track movement patterns, activity levels, and changes in behavior, which are often the first indicators of illness in animals. For example, a decrease in activity or abnormal movement may signal pain or lethargy, which could be caused by a variety of conditions, such as gastrointestinal issues or infections (25). AI systems, by analyzing this data, can identify patterns that are associated with certain health problems, making it easier for farmers to spot potential concerns early, even when animals show no outward signs of illness.

AI-driven wearable health monitoring systems can also incorporate data from other sources, such as environmental conditions and farm management practices. For example, integrating weather data, such as extreme temperature changes or humidity levels, with health data can help to predict or explain health issues (16, 20, 21). In some cases, environmental factors such as cold weather or high humidity can exacerbate conditions like respiratory infections or stress in rabbits (1, 3, 4). AI models can analyze these factors in real-time and provide farmers with a holistic view of what might be influencing the health of their rabbits, helping them adjust farm conditions to minimize stress and maximize animal welfare (9). This integrated approach provides a level of proactive management that traditional methods lack, allowing farmers to prevent health issues before they become critical.

Moreover, continuous health monitoring through wearables can improve herd management by providing valuable longitudinal data. Over time, AI systems can analyze the trends and patterns in the rabbits’ vital signs, identifying any long-term health changes or gradual declines in wellness (21). This can be crucial for managing the health of individual animals, as well as for assessing the overall health trends within a farm’s rabbit population. Such insights can guide breeding programs, early disease detection, and personalized care for individual rabbits, ultimately improving both the health and productivity of the farm (19, 21). By continuously collecting and analyzing health data, farmers can make more informed decisions, leading to better outcomes for both the animals and the farm’s operational efficiency.

Finally, the integration of wearable sensors with AI can help reduce the need for frequent manual checks on the rabbits, saving farmers time and labor costs. With real-time alerts, farmers can prioritize interventions, addressing health issues only when necessary, rather than conducting routine checks that may not yield significant results. This shift toward automated health monitoring optimizes farm management, ensuring that rabbits are receiving the best possible care while also improving the efficiency of farm operations (10). As the technology continues to evolve, it promises to provide even more detailed insights into the health of rabbits, further enhancing the welfare of animals in commercial farming systems.

## 6 AI in monitoring rabbit behavior

Artificial intelligence (AI) offers a range of applications in monitoring rabbit behavior, playing a vital role in assessing welfare, social interactions, and maternal care. One key application is AI’s capacity to analyze and interpret behavioral patterns to evaluate rabbit welfare (19, 21). By continuously tracking activities such as feeding, resting, grooming, and movement, AI systems can identify signs of stress, discomfort, or abnormal behavior that may signal underlying health problems or environmental stressors. This continuous behavioral surveillance enables timely and informed interventions, helping to ensure that rabbits are maintained in environments conducive to their well-being. Beyond individual welfare assessment, AI technologies are increasingly used to monitor social interactions within rabbit groups. By observing patterns of interaction, AI can reveal insights into social dynamics such as dominance hierarchies, group cohesion, and compatibility. These insights are essential for optimizing group management, reducing conflict, and minimizing stress related to poor social integration. Additionally, AI plays an important role in monitoring maternal behaviors. AI can detect and analyze key maternal actions such as nest building, grooming, nursing, and interactions between does and their kits. By identifying early signs of neglect or difficulties in maternal care, AI systems can prompt timely interventions to support both the mother and her offspring. This holistic approach to behavioral monitoring enhances the overall management of rabbit populations, promoting improved health, welfare, and productivity.

### 6.1 Behavioral analysis for welfare

AI-driven behavioral analysis systems use a combination of video cameras, motion sensors, and wearable devices to monitor rabbits’ activity levels, feeding behavior, grooming patterns, and social interactions. These technologies process vast amounts of behavioral data to identify early signs of distress, illness, or discomfort, allowing for timely interventions and more effective care (10, 16). AI systems also enable real-time monitoring of large groups of rabbits, making them especially valuable in commercial farming environments where manual observation is less feasible.

For example, AI can analyze video footage to detect subtle behavioral changes such as reduced movement or altered feeding habits, early indicators of illness or environmental stress. Additionally, AI can identify aggressive behaviors or signs of social exclusion, which are associated with injury or emotional distress (17). Beyond physical behaviors, AI algorithms can assess emotional responses by detecting signs of stress, such as rapid breathing or abnormal movement patterns. These behavioral responses can be correlated with environmental stressors, such as temperature fluctuations, overcrowding, or inadequate ventilation, allowing farmers to make informed adjustments to improve living conditions.

One of the major strengths of AI-based behavioral analysis is its ability to integrate multiple data streams to provide a holistic understanding of rabbit welfare. By combining behavioral data with environmental sensor readings and physiological metrics from wearable devices, AI systems can uncover patterns that indicate poor welfare conditions. For instance, if a rabbit exhibits stress-related behavior, AI can correlate this with elevated ambient temperature or humidity levels. These actionable insights allow farmers to proactively manage their herds and mitigate potential welfare risks before they escalate (13, 16, 21).

AI-enabled systems can predict discomfort or health issues before they become severe by continuously learning from new data, refining their predictions, and enabling precise interventions. This feedback loop enhances both animal welfare and farm productivity. Additionally, AI-powered computer vision platforms can detect physical indicators of well-being, including changes in posture, body condition, and movement patterns, which may indicate health status or emotional state. Algorithms can also assess facial expressions and micro-movements to further improve welfare assessment accuracy (20, 21). As these technologies advance, they are expected to become even more sensitive to subtle behavioral changes, providing earlier and more accurate alerts for emerging issues. The integration of AI in rabbit health and welfare management is revolutionizing how farms monitor and care for animals. Technologies such as behavioral analysis, predictive modeling, and health monitoring systems give farmers powerful tools to detect early signs of illness, discomfort, or poor environmental conditions (7, 9, 13). These tools enable timely, evidence-based interventions that enhance welfare standards and operational efficiency. Moreover, AI not only supports physical health but also helps ensure emotional well-being by identifying and mitigating environmental stressors.

As AI systems continue to evolve, they will become indispensable tools for sustainable and ethical rabbit farming. With continuous learning and real-time monitoring, these technologies enhance decision-making, reduce labor intensity, and contribute to a more humane and productive farming system. Ultimately, the ongoing development and adoption of AI in animal husbandry promise a future where animal welfare and agricultural efficiency go hand in hand, benefiting animals, farmers, and the wider agricultural sector alike (20, 21).

### 6.2 Monitoring social interactions of rabbits using AI

Rabbits are highly social animals whose interactions within a group influence not only their emotional states but also their long-term health, reproduction, and productivity. In intensive farming environments, where many rabbits are housed together, monitoring social behaviors is critical to maintaining welfare standards. Artificial intelligence (AI), particularly through machine learning and computer vision, offers an innovative approach to tracking and interpreting these complex social dynamics (15, 26).

AI technologies can analyze video footage and sensor data to identify and classify various types of social interactions, such as grooming, playing, submission, and aggression, without the need for continuous human observation (15, 27). Unlike traditional manual methods, AI systems can automatically recognize and quantify these behaviors in real-time, even in large populations. This capability is particularly valuable for detecting problematic behaviors like persistent aggression, bullying, or social withdrawal, which are often early indicators of poor welfare or environmental stressors (15, 26, 27).

Recent studies have demonstrated the potential of AI-driven monitoring to improve group management in rabbit farming. For instance, algorithms can track individual animals within a pen to identify abnormal interaction patterns, such as repeated aggressive encounters or avoidance behavior, that suggest deteriorating social conditions (15, 27). These insights allow for timely interventions, including regrouping animals, adjusting stocking density, or modifying housing design to reduce conflict and improve compatibility.

AI systems have also been used to detect stress-related social behaviors, such as excessive grooming or pacing, which may not be immediately apparent to the naked eye. These behaviors often correlate with environmental deficiencies, such as poor ventilation or overcrowding. In one study, AI-based tools successfully linked increased aggression to specific environmental stressors, enabling farmers to make targeted improvements that enhanced rabbit welfare (15, 27).

By focusing specifically on the analysis of social interactions, AI supports a more nuanced understanding of rabbit group behavior, complementing broader welfare monitoring tools. Rather than replacing traditional methods, these technologies enhance farmers’ ability to respond promptly to behavioral cues, ultimately promoting healthier social environments and reducing the risk of injury or stress-related illness in group-housed rabbits.

### 6.3 Maternal behavior and kit interaction

Maternal behavior in rabbits is critical to the survival and development of their offspring. Female rabbits (Does) exhibit specific behaviors such as grooming, nursing, and protecting their kits, which are essential for the kits’ physical and emotional well-being. AI technologies can play an important role in monitoring these behaviors to ensure that the maternal care provided is adequate and consistent. For instance, AI systems can track the frequency and duration of nursing sessions, the grooming habits of the doe, and interactions between the mother and her kits. This real-time tracking can provide early indications of any problems, such as maternal neglect, that could negatively impact the kits. By identifying these issues promptly, farmers can intervene to provide the necessary support, ensuring that the kits receive adequate care and nourishment (16, 28, 29).

One of the key aspects of monitoring maternal behavior is tracking the timing of nursing and other mother-young interactions. González-Mariscal and colleagues (30) highlighted the importance of the timing of suckling in rabbits. The frequency and regularity of suckling affect both the mother’s milk output and the duration of contact between the doe and her kits. If AI systems can track suckling patterns, they can help identify deviations from normal behaviors, such as irregular nursing intervals or insufficient milk production. This allows for timely interventions to optimize the kits’ nutrition and growth, ensuring the offspring thrive in the early stages of life.

AI-based systems can also monitor other maternal behaviors, such as grooming, which has significant implications for both the physical and psychological health of the kits. Grooming not only helps maintain cleanliness but also serves as a bonding activity between the doe and her young. The absence or reduction of grooming behaviors could indicate stress or maternal neglect. AI can detect changes in grooming patterns and flag any anomalies that might require human intervention. This can prevent the onset of stress-related issues that might affect the kits’ health and development (31–36).

The ability of AI to monitor the mother’s protective behaviors is another crucial aspect. In the wild, rabbits use complex nesting behaviors to protect their young from predators and environmental factors. Bilkó et al. (16) explored the composition of wild rabbit nests and how these natural behaviors could inform the breeding practices of domestic rabbits. AI can assist in monitoring the doe’s protective behaviors by tracking her movements and interactions with her nest. By analyzing these data, AI can help farmers ensure that does are providing sufficient protection and security for their kits, preventing them from being exposed to harmful conditions or predators.

Moreover, AI technologies can provide insights into how environmental factors influence maternal behavior. For example, the presence of stressors such as overcrowding, inappropriate temperatures, or inadequate nesting conditions can affect a doe’s maternal instincts and behaviors. Caba et al. (29) emphasized the significance of environmental factors in expressing maternal care in rabbits. By utilizing AI to track both the behavior of the doe and the environmental conditions, farmers can adjust the living space and conditions to support maternal behaviors better. Such adjustments could improve both the well-being of the doe and the kits, resulting in healthier offspring and better productivity in farm settings.

Lastly, the use of AI in monitoring rabbit maternal behavior provides an opportunity to enhance the welfare of rabbits in breeding and commercial environments. The ability to continuously monitor behaviors without human bias allows for a more objective and efficient approach to ensuring maternal care. AI-driven systems could enable a more humane and scientifically informed approach to managing rabbit reproduction and care. By identifying issues early, such as stress or insufficient maternal behaviors, interventions can be made before they lead to more serious problems, improving the welfare of the animals and the success of breeding programs (32). Integrating AI into maternal care monitoring represents a significant step forward in the ethical management of rabbit breeding and care.

## 7 Challenges and future directions

### 7.1 Data collection and integration

The successful implementation of AI in rabbit husbandry relies heavily on the availability of high-quality, reliable data. For AI to be effective in monitoring and improving the welfare of rabbits, it is essential to collect accurate and comprehensive data from various sources. These sources often include wearable devices that track the movements and behaviors of the rabbits, cameras that capture visual data of social interactions and maternal behaviors, and environmental sensors that monitor temperature, humidity, and light levels. By integrating data from these diverse sources, AI models can be trained to recognize patterns in rabbit behavior, detect stress indicators, and optimize environmental conditions. However, to fully realize the potential of AI in this domain, ensuring the consistency and reliability of the data is critical. Without accurate data, AI models risk producing inaccurate predictions or insights, which could ultimately harm animal welfare.

One of the primary challenges in integrating data from different sources is ensuring consistency and accuracy. Wearable devices, for instance, may produce data with varying degrees of precision depending on the type of sensor used, the quality of the device, or the animal’s specific conditions. Cameras and visual recognition tools, on the other hand, can face challenges such as lighting issues, occlusions, or the complexity of accurately identifying individual rabbits within a group. Environmental sensors may also face calibration issues or fail to account for all variables that affect the animals’ well-being. These variations in data quality can make it challenging to combine and analyze the data in a meaningful way. Therefore, careful calibration and validation of each data source are essential to ensure that the information being fed into the AI models is both accurate and reliable.

A major hurdle in overcoming these challenges is the need for standardization of data formats and communication protocols. Different types of sensors and devices often use proprietary formats or systems, which can create barriers to the seamless integration of data. Standardized protocols would enable data from wearables, cameras, and environmental sensors to be harmonized and combined effectively for AI analysis. This standardization would also facilitate the development of interoperable systems across different farms, allowing farmers and researchers to collaborate more effectively and share insights from their AI-based monitoring systems. Additionally, such standards would make it easier to scale AI solutions, ensuring that technological advancements can be widely adopted across the industry. For these reasons, establishing clear guidelines for data formats and integration protocols will be crucial for realizing the full potential of AI in rabbit husbandry and animal welfare management.

### 7.2 Ethics and welfare considerations

While AI technologies offer significant benefits for improving rabbit health and welfare, they also raise important ethical considerations that must be addressed. One major concern is the potential impact of continuous monitoring on the animals. For AI systems to effectively track behaviors and environmental conditions, sensors and cameras must be placed in the rabbits’ living spaces, which could lead to increased stress or discomfort if the animals are sensitive to the presence of these devices. The process of attaching wearable sensors, for example, could be invasive or cause distress, particularly if the devices are poorly designed or improperly fitted. Moreover, the presence of cameras and other monitoring tools may create a sense of unease in the rabbits, affecting their natural behavior. As noted by Siddiqui et al. (33), while technological advancements such as AI can bring positive changes to production systems, they must be weighed against potential animal welfare risks. Ethical considerations must guide the development of AI applications, ensuring that any technology used does not contribute to harm. The balance between welfare and monitoring needs is critical to avoid unintended consequences that might undermine the aim of improving rabbit care.

Careful consideration of animal welfare should also include minimizing the disruption caused by monitoring technologies. AI systems, while helpful, should be implemented to improve the rabbits’ living conditions rather than exacerbate stress or discomfort. For example, AI-based monitoring could be adjusted to ensure the monitoring devices are as unobtrusive as possible, allowing for effective data collection without intruding on the animals’ natural behaviors. Dalmau et al. (34) emphasized that when assessing animal welfare in production systems, any monitoring should be done with consideration for the animal’s ability to express natural behaviors. Additionally, the use of AI should aim to enhance the overall environment for the rabbits, addressing issues such as overcrowding, poor ventilation, or inadequate space factors that could negatively affect their well-being. Temple and Manteca (35) also highlighted that welfare concerns in production systems need to be met with practical solutions, and AI can be an important tool in identifying and addressing these environmental stressors.

Several studies have explored the use of AI and digital technologies in rabbit monitoring, highlighting both the benefits and ethical implications. For instance, Irawati et al. (36) developed an Android-based expert system for diagnosing rabbit diseases, demonstrating how digital platforms can aid in veterinary care, though the integration of such tools into daily management must consider animal comfort. Similarly, Lai et al. (37) introduced a rabbit pregnancy diagnosis device that relies on optical sensing, emphasizing accuracy and efficiency, but also implying the need for minimally invasive methods. Zhang et al. (38) used deep neural networks to predict sudden death in rabbits under exhaustive exercise, raising questions about the ethical design of testing environments and their impact on animal welfare. Zhang and Qian (39) proposed intelligent fault identification in farm environments using neural network models, stressing automation benefits while also highlighting the need for humane system integration. Moreover, predictive models like those discussed by Horwitz et al. (40) illustrate the power of machine learning in anticipating clinical outcomes, yet underscore the responsibility to use these tools without compromising ethical standards. Establishing guidelines for the ethical use of AI in animal husbandry is crucial to maintaining the balance between technological advancement and animal rights. Such measures would ensure that the benefits of AI are fully realized while preventing potential harm to the rabbits being monitored. Ultimately, the welfare of the animals should always remain a top priority, with AI acting as a tool to enhance their quality of life rather than compromise it.

### 7.3 Future research directions

Future research in AI applications for rabbit husbandry should prioritize improving the accuracy, adaptability, and overall applicability of these systems to enhance animal welfare and farming practices. One important direction is the integration of AI with IoT technologies to create a more holistic monitoring system. By combining AI with IoT, sensors, and devices can communicate in real-time, enabling continuous tracking of the rabbits’ health, behaviors, and environmental conditions. These technologies can provide a more dynamic understanding of the rabbits’ needs, allowing farmers to make data-driven decisions to optimize care. Moreover, ensuring that AI systems are capable of functioning effectively across diverse environmental conditions ranging from controlled indoor environments to varying outdoor climates will be essential for AI’s broader implementation in rabbit farming. IoT devices can also monitor environmental factors like temperature, humidity, and ventilation, which are key to ensuring the rabbits’ well-being. As farms differ in size, layout, and location, systems capable of adapting to these variables will provide more tailored and relevant solutions.

Another crucial area for development in AI for rabbit husbandry is the personalization of care through adaptive AI models. Just as human healthcare increasingly takes a personalized approach, AI systems for rabbit welfare should be designed to consider the unique characteristics of each animal, such as breed, age, health status, and individual behavioral traits. For instance, AI could monitor the development of young rabbits differently from adult rabbits, considering their nutritional needs, growth patterns, and maternal interactions. Similarly, AI could be programmed to account for specific health issues, such as common diseases or injuries in certain rabbit breeds, and alert farmers when personalized care is needed. This level of precision would enhance the overall welfare of individual animals, ensuring they receive the care and attention required at different life stages or in response to specific health challenges. By incorporating these personalized elements into AI models, rabbit farming could evolve into a more species-specific and humane practice, where each rabbit’s well-being is continuously monitored and addressed based on their unique needs.

Furthermore, the ongoing development of AI systems for rabbit husbandry must also emphasize the need for scalability and ease of implementation. While cutting-edge AI technologies and IoT integration are promising, they must be accessible and practical for farmers, particularly those in smaller or more traditional farming operations. Research should explore ways to make AI tools cost-effective, user-friendly, and adaptable to different scales of operation, ensuring that even farms with limited resources can benefit from advanced monitoring technologies. The creation of standardized AI platforms, as well as user-friendly interfaces, would make these technologies more accessible and easier to implement, regardless of the farm’s size or technological infrastructure. As these systems become more accessible, widespread adoption of AI in rabbit husbandry can become a reality, offering long-term improvements in animal welfare, farming efficiency, and sustainability.

**Figure 5:**
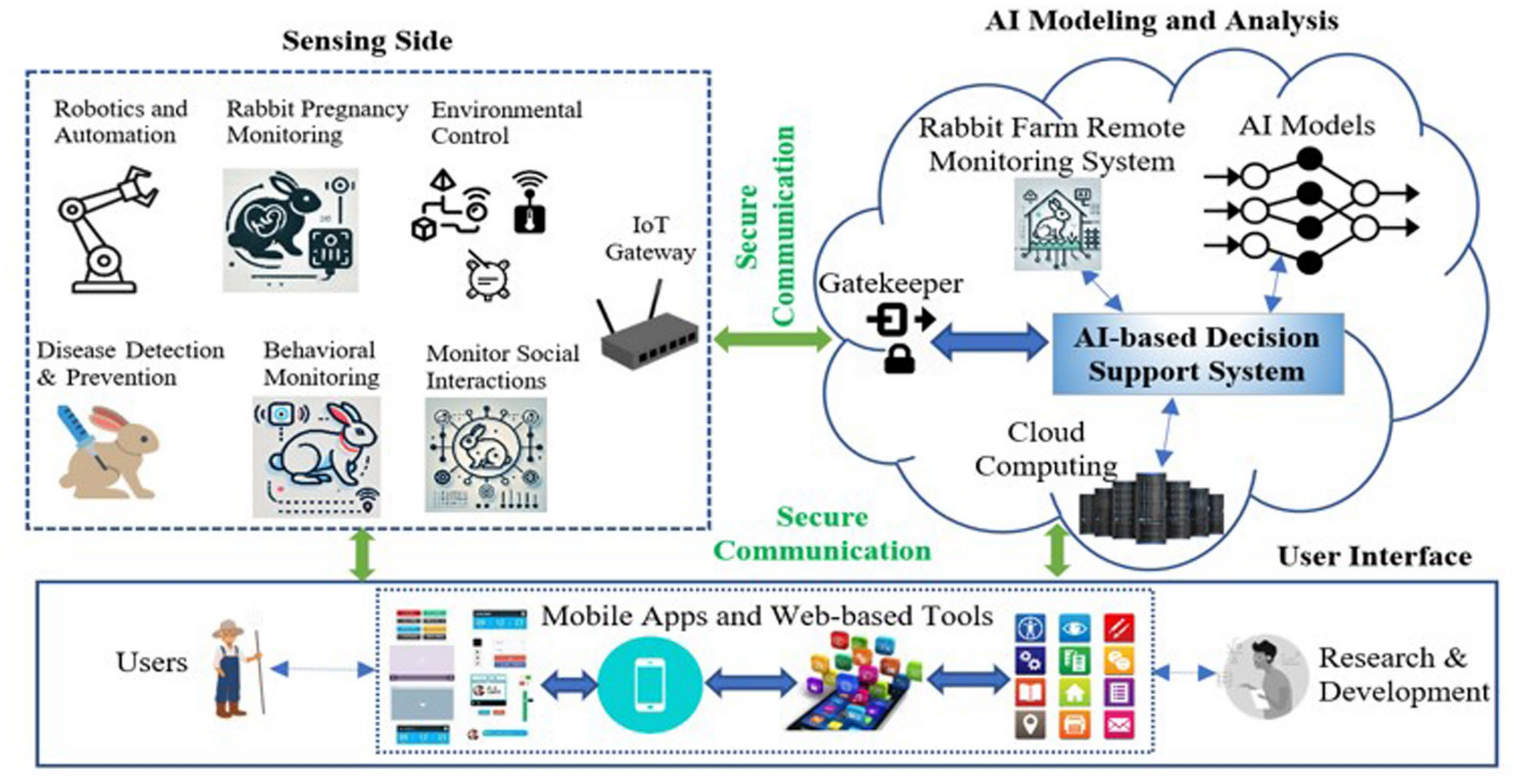
Architecture of a proposed AI-powered system for next-generation rabbit farming. It presents an integrated framework combining sensors, machine learning, and real-time data analytics for precision rabbit management.

There is a persistent need to develop an innovative AI-powered system for next-generation rabbit farming. Figure 5 shows the runtime environment for the proposed AI-based system. On the sensing side, data is collected from various IoT end-devices embedded within AI applications in the rabbit farm, including robotic and automation, pregnancy monitoring, environmental control, disease detection and prevention, behavioral monitoring, and social interaction monitoring. IoT gateways will be used to aggregate these sensing data and coordinate the connectivity of the IoT devices to each other and the cloud side.

On the cloud-based AI modeling and analysis side, the proposed AI-based Decision Support System (DSS) receives the aggregated events from different sensing sites through the Gatekeeper, which implements various security mechanisms to ensure that the incoming messages are legitimate. The aggregated events are then stored in distributed databases on the cloud side. The platform also sends a set of parameters to gateways advising them on how to detect events, construct their messages, and how often to send them (once or periodically, how frequently, etc.). The remote monitoring system generates synthetic data to fulfill specific needs or conditions that may not be found in the original real data from the sensing sites. Both natural and artificial data are used to train various ML models such as Convolutional Neural Networks (CNNs), Recurrent Neural Networks (RNNs), Deep Neural Networks (DNNs), and Deep Belief Networks (DBNs).

System users (e.g., farmers) and students can quickly initiate and manage IoT applications using a web-based Graphical User Interface (GUI) from their personal computing devices, such as personal computers and smartphones. An application is triggered by a user who uses the GUI to send a request to the AI-enabled DSS to create a new IoT application. For example, consider an intelligent poultry farming application running on top of the proposed AI-powered system, which enables farmers to monitor farm conditions from anywhere with the help of sensors and automated feeding and behavior monitoring. Different sensors will be used to measure environmental parameters according to the bird’s growth requirements, allowing for controlled environmental conditions on the farm. The GUI will help users access the deployed AI system remotely. It eliminates the need for constant manual monitoring. This design provides cost-effective and optimal solutions to farmers with minimal manual intervention.

## 8 Conclusion

AI has the potential to revolutionize the management of rabbit pregnancy, delivery, health, and behavior by offering non-invasive, real-time monitoring tools that enhance efficiency and improve animal welfare. Through the use of AI-driven sensors, machine learning models, and computer vision systems, farmers can more accurately predict pregnancies, manage labor, monitor health, and detect behavioral changes. While challenges related to data integration and ethical concerns remain, the future of AI in rabbit farming looks promising. Continued research and development will be crucial in unlocking the full potential of AI technologies to improve rabbit husbandry practices and ensure better outcomes for both farmers and rabbits.
